# Attitudes of Michigan Female College Students about Pharmacists Prescribing Birth Control in a Community Pharmacy

**DOI:** 10.3390/pharmacy8020099

**Published:** 2020-06-09

**Authors:** Mary Beth O’Connell, Leah Samman, Teresa Bailey, Larissa King, Gregory S. Wellman

**Affiliations:** 1 Eugene Applebaum College of Pharmacy and Health Sciences, Wayne State University, Detroit, MI 48201, USA; leah.vader@wayne.edu; 2 College of Pharmacy, Ferris State University, Big Rapids, MI 49307, USA; teresabailey@ferris.edu (T.B.); kingl16@ferris.edu (L.K.); wellmang@ferris.edu (G.S.W.); 3 School of Medicine Health Clinics, Western Michigan University Homer Stryker, Kalamazoo, MI 49008, USA

**Keywords:** birth control, hormonal contraception, community pharmacy, pharmacy access, pharmacist prescribing, pregnancy, college students

## Abstract

In the United States, the overall unintended pregnancy rate is about 45%. Women between 20–24 years old account for 59% of the unintended pregnancies. Continuous birth control use is related to decreasing unintended pregnancies. Therefore, we assessed female college students’ opinions about pharmacists prescribing birth control in a community pharmacy using an intersectionality framework. A survey with 49 items about provider attributes, pharmacy services use and evaluation, advantages and barriers of pharmacists prescribing birth control, sexual and reproductive history, and demographics was distributed by survey link and QR code. Recruitment was done by investigators and students (snowballing technique) via emails, social media posts, and direct student contact. Respondents (*n* = 859) were 23.0 ± 4.9 years old, 83% white, 64% healthcare students, 32% student pharmacists, 69% sexually active, 68% with at least one episode of unprotected intercourse within a year, and 29% never using condoms. Forty-six percent of students were extremely likely and 26% moderately likely to get birth control from a pharmacist because it would be easier to adhere to birth control, could prevent unintended pregnancies, would be more convenient, and require less time. Concerns included the lack of Pap screenings and prescriptions written for the wrong birth control. Within most student characteristics or attitudes assessed, at least 70% of the students would use this service. Based on student opinions, female college students would use pharmacists prescribing birth control services.

## 1. Introduction

Unintended pregnancies are recognized as individual, family, and societal concerns. Forty-five percent of pregnancies are unintended in the United States with higher prevalence in those 20–24 years old, with low income, and/or from minority groups [1]. The cost of unintended pregnancies was estimated to be $11.1 billion dollars per year [2]. Of the unintended pregnancies, 49% of the mothers give birth, 36% have an elective abortion, and 15% have a miscarriage [3]. Prior to an abortion, 54% of the women were not using contraception and 29% had wanted to use birth control but experienced barriers to obtaining it [4]. About 19 million women of childbearing age live in areas without federally funded facilities that prescribe birth control [5]; however, many of these areas would have a community pharmacy. Other barriers include cost and insurance coverage of contraception, payment of contraception prescribers and family planning clinics, religious beliefs limiting access and prescribing, insufficient education on contraception methods, privacy of use, requirements for a full women’s health screening visit, and health care inequities [6].

Both the Healthy People 2020 initiatives [7] and the Centers for Disease Control and Prevention (CDC) [8] have goals and plans to decrease unintended pregnancies. The Healthy People 2020 goal was to increase the proportion of pregnancies intended from 51% to 56% [7]. The CDC goal is to build capacity for greater access to contraception services, but they do not specifically define the services to accomplish this goal [8]. Historically the focus for prevention has been on abstinence instead of contraception, even though no evidence exists that these programs increase the age of first sexual intercourse nor decrease number of sexual partners [9,10]. Based on federal funding of contraception through the Title X family planning program, 1.9 million unintended pregnancies were estimated to be averted in 2014 by increasing low cost birth control options and making contraception access easier [3]. Increased use of birth control and decreased or delayed sexual intercourse were attributed to decreasing unintended pregnancies in adolescents [11]. Some women (29%) who have had an abortion stated they had desired birth control use but did not because of cost, adverse reaction concerns, lack of knowledge of where to get birth control, lack of transportation, and desire not to see a physician [4]. After the abortion, 76% of the women wanted to use birth control with 52% preferring contraceptive pills. Many women have stated that the requirement for a prescription for oral contraceptives has proven to be a barrier for its use [2,12,13]. Two models have been proposed to help decrease unintended pregnancies by giving women greater access to contraceptives, which are changing birth control to over-the-counter status [2,12] and having pharmacists prescribe birth control [12,14,15,16,17].

The American College of Obstetricians and Gynecologists (ACOG) position on this topic is that hormonal contraception should be available over the counter with no age restrictions [2,12]. ACOG is working at the federal level to obtain a Food Drug Administration reclassification of this medication via legislative change. Some concerns with this model are reduced women’s health screenings, contraceptives unsafe for self-selection, and over-the-counter contraception costs could be prohibitive and/or not covered by insurance plans. As an intermediary step, ACOG has acknowledged and approved the pharmacist prescribing model, which requires state level legislative changes, until over-the-counter contraception status is legally approved [12].

The pharmacist prescribing model allows pharmacists in community pharmacies to prescribe hormonal contraception for patients. Pharmacists obtain this ability through legislation or collaborative practice agreements with physicians. Eleven states and the District of Columbia have scope of practice or standing order legislative authorities allowing pharmacist to prescribe hormonal contraception [14,15,16,17]. Another model is through collaborative practice agreements, such as Washington’s authority for prescribing birth control. Recently (March 2020), one large pharmacy chain in Michigan also implemented a collaborative practice agreement for their pharmacists to prescribe birth control to adult women. Additional states have pending legislation.

Data have documented adolescents [18,19,20], women [18,20,21], pharmacists [22], student pharmacists [23] and primary health care providers [24] support of pharmacists prescribing birth control. More information is needed to identify which teens and women will use pharmacists for their birth control access. In California, 90% of pharmacists agreed with community pharmacists prescribing birth control, and 63% stated willingness to add this service to their practice [22]. Seventy-six percent of reproductive health physicians and 70% of midlevel practitioners supported pharmacists prescribing birth control, higher approvals than behind the counter and over-the-counter birth control access [24].

Emerging outcomes from pharmacist prescribing birth control are positive. Within two years of implementing pharmacist prescribed birth control for Oregon women receiving Medicaid, an estimated 51 unintended pregnancies were prevented, and about $1.6 million dollars saved [17].

Public opinions and perceptions are important in advancing pharmacists as birth control prescribers through state legislative processes and approvals. Gaps in identifying characteristics associated with using pharmacist birth control services still exist or need confirmation. The viewpoints of women aged 20–24 are particularly important since they have the highest prevalence of unintended pregnancy in the United States [2]. Therefore, the purpose of this study was to determine female college students’ opinions about and willingness to use pharmacists for obtaining hormonal contraception in a community pharmacy across a wide range of student characteristics, attitudes, and health service opinions.

## 2. Methods

### 2.1. Design

**Figure 1:**
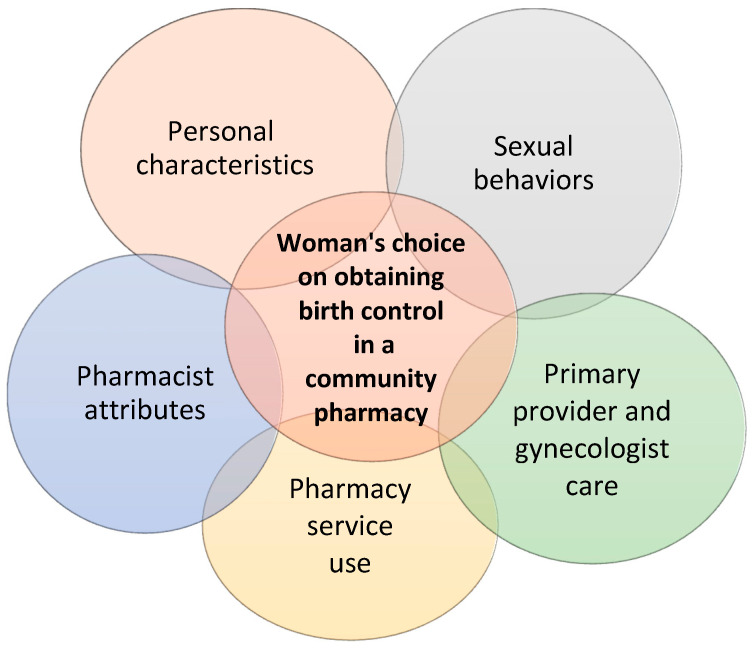
Influences on Choosing a Pharmacist to Prescribe Birth Control.

Investigators developed a survey that was distributed to a convenience sample. We chose an intersectionality approach by querying about characteristics, attitudes, sexual behaviors, and health services use and opinions (Figure 1) that overlap to influence a woman’s health decision making. The research project was deemed exempt via category 2 criteria (because identity of the students would not be ascertained from the data and no criminal or civil liability or student damage could be determined from results) by two university institutional review boards. An assent information form was included as the first question of the survey.

### 2.2. Participants

Female college students of any age from any college in any curriculum were eligible to participate. Students selecting intersex as their gender were included but male students were excluded. Surveys needed to have at least one other item besides gender answered to be included. Surveys from students residing or attending college outside of Michigan were excluded.

Recruitment was primarily conducted at two universities and two community colleges. These educational institutes were chosen to gather student opinion throughout the whole state and provide metro, urban, and rural geographical variability. One university was in Detroit, one community college in a Detroit suburb, one university on the west side of the state, and one community college in the upper peninsula.

### 2.3. Survey Development

A validated survey related to our project was not available. Literature was reviewed related to over-the-counter birth control, emergency contraception, pharmacists prescribing contraception, obstetrics and gynecology practice/provider patient preferences, pharmacy clinical services in community pharmacies, and position statements related to these topics to create our survey. Survey items were developed based on published non-validated survey items or newly created to assess this study’s purposes. A 46-item survey (Appendix A) was initially developed with two additional items (i.e., college attending and current city of residence) added after the study started. The survey topics (in survey order) were assent (1 item), health care experience attributes of gynecologists or clinic providers and future pharmacists prescribing birth control (2 items with 9 sub-items each), pharmacy services and evaluation (3 items), pharmacist prescribing birth control advantages and barriers (6 items), demographics (21 items), and sexual and reproductive history (15 items). For the health care experience attributes section, students were presented with two scenarios where she obtained a birth control prescription from a gynecologist or primary care provider and one from a community pharmacist with the ability to prescribe birth control. Students were then asked to rate their perception of nine provider attributes for both scenario providers on a scale of one (lowest) to ten (highest). Students could skip answering any of the items. No personal identification information was collected. A second survey was created for the raffle of two $250 gift certificates. The survey platform was Qualtrics.

The survey was distributed to ten students from the two universities for review of item clarity and understanding, and survey completion time. Suggestions were incorporated into the final survey.

### 2.4. Survey Distribution

The Qualtrics survey URL link was used and later the survey QR code was added to advertisements. One university posted an advertisement two times a week in a daily newsletter sent out to every student over a span of six weeks. They also sent a mass email containing the survey link to all college of pharmacy students at this university. At the other university, a study notice or flyer was posted in student news feeds, research recruitment webpage, campus housing, Facebook pages and other social media. Both universities sent emails to female student organizations asking for them to email or post the advertisements. Some in class pharmacy and other program announcements were made. Students were also asked to participate while dining in the university commons area. One community college sent out broadcast emails to the whole student population. The other community college sent advertisement to one curriculum, i.e., nursing, for which a student mass email list existed. Students participated in snowballing techniques by sending emails to their female college friends or posting on their own social media sites. Recruitment occurred from April 2019 to February 2020. Target goal of 1500 completed surveys was not achieved.

### 2.5. Analysis Plan

Descriptive statistics with inferential statistics, Chi square, and ordered logistic regression were used to analyze the data. For Chi square analyses, five-point Likert scales were collapsed to three-point Likert scales. For likelihood of obtaining birth control from a community pharmacist, those stating no birth control use were excluded from crosstab analysis. Extremely and moderately likely responses were collapsed to likely, not sure responses remained, and not very likely and not likely at all responses were collapsed to not likely. These analyses were done with IBM SPSS Statistics version 26.0 (IBM Corp., Armonk, NY, USA). For the ordered logistic regression, the full five-point Likert scale and the students not using birth control were included. The pharmacist attributes were on a scale of one being low to ten being high. This analysis was run on 816 of the respondents using listwise deletion. The ologit module in Stata 16.0 was used for this analysis. A *p*-value of less than or equal to 0.05 was considered significant.

## 3. Results

### 3.1. Survey Participants

Because of the survey distribution techniques, total sampling cannot be quantitated and therefore a response rate cannot be calculated. The survey was opened by 1110 students with 859 (77.4%) surveys included in the analysis. The reasons for survey deletions were 8 surveys had no responses, 73 were started by male students, 153 had no answers except for gender (1 intersex, 152 women), 11 were completed by female students attending non-Michigan colleges, and 6 were completed by female students living outside of Michigan. The average age and standard deviation of the participants was 23.0 ± 4.9 years (range 17–56 years old). The student participant characteristics and associated likelihood to obtain birth control from a pharmacist are in Table 1.

### 3.2. Student Perceived Advantages and Disadvantages of a Pharmacist Prescribing Birth Control

Overall, female college students stated they would obtain birth control from a pharmacist with most students stating they were extremely likely (46.3%) to do so, followed by 26.3% moderately likely, 12.7% unsure, 8.7% not very likely, and 3.3% not likely at all. The mean response and standard deviation was 2.1 ± 1.3 [six-point scale with one (extremely likely) to six (never)].

Students had positive comments about pharmacists prescribing birth control but also some concerns. Survey advantages and concerns prompted responses (survey items) are listed in Table 2. The mean number of prompted and other stated advantages was 4.8 ± 1.6 with most students listing five (27.4%) or six (31.7%) advantages. Additional advantages included a Pap or pregnancy test not needed (3), insurance coverage checked (3), birth control switching easier (3), less stress (2), blood pressure checked (1), fewer appointments (1), pharmacists more knowledgeable (1), pharmacists more available for questions (1), easier to get in an emergency (1), easier to get refill (1), and easier to get answers (1). Five students (0.6%) listed no advantages.

The mean number of prompted and other stated concerns were 2.1 ± 1.3 with most students listing one (34.3%) or two (24.9%) concerns (Table 2). Additional concerns included no medical chart access (16), pharmacists are overworked (12), relationship with physician needed (10), lack of privacy (8), less education than other providers (7), pharmacists need more birth control education (6), insufficient patient education provided (5), lack of insurance coverage of visit (2), should be pharmacist and doctor team (1), limited birth control options to be prescribed (1), pharmacist not able to resolve side effects (1), pharmacist refusal to dispense (1), non-hormonal options needed (1), less professional environment (1), lack of documentation (1), and uncomfortable with pharmacists prescribing (1). Forty-eight students (5.7%) listed no concerns.

### 3.3. Student Characteristics and Birth Control from a Community Pharmacist

In Table 1, the percent of students stating they were extremely or moderately likely to get their birth control from a pharmacist in a community pharmacy is displayed. Only citizenship, degree program, health professional student status, and type of health professional student had significant differences within the category. Sixty-seven to 77% of the various religious/spirituality groups would obtain birth control from a pharmacist. One third of the students would use birth control even if their religion opposed this medication. One percent stated because their religion did not support birth control, they would not use this medication. For 4.7% of the students, they stated their religion only approved birth control if married, and 1.7% stated permission to use birth control only after marriage and with partner approval. Almost a third of the students (31.1%) had no beliefs that would impact birth control use. Some students (27.7%) had religions that did not oppose birth control use.

### 3.4. Sexual Behaviors and Likelihood of Obtaining Birth Control from a Pharmacist

Sexual practices of the students and likelihood of obtaining birth control from a pharmacist results are presented in Table 3. Many students (68.8%) were in a sexually active relationship with a man and 3% with a woman. About 14% of the students stated they did not need birth control because they were not sexually active (9.5%), or were incapable of getting pregnant (1.6%), pregnant (0.3%), or postmenopausal (0.3%). Nineteen students (2.2%) practiced abstinence. Prescription or provider administered birth control used by the students were 49% pills, 9.1% IUDs, 3.4% implants, 2.6% shots, 2.1% emergency contraception, and 2.0% rings. Of those using hormonal birth control, most students had only one indication (42.8%), with 41.3% having two indications, and 15.9% having three indications. The most common indication for birth control was contraception (53.6%), followed by menstrual disorders (38.8%), acne (18.5%), and other (3.7%).

For the specific sexual practice variables, 69% to 89% of the female college students stated they were extremely or moderately likely to get their birth control from a pharmacist (Table 3). Only sexually active with a man (*p* = 0.027) and times of unprotected intercourse in the last year (*p* = 0.044) had significant differences within the variable.

An ordinal regression on likelihood of obtaining birth control from a pharmacist did not yield a significant model for the variables age, age at first intercourse, number of sexual partners, or number of times intercourse per week (model significance *p* = 0.7413).

### 3.5. Attitudes and Perceptions of Pharmacists and Pharmacist Birth Control Healthcare Services

#### 3.5.1. Pharmacy Services and Birth Control from a Community Pharmacist

**Figure 2:**
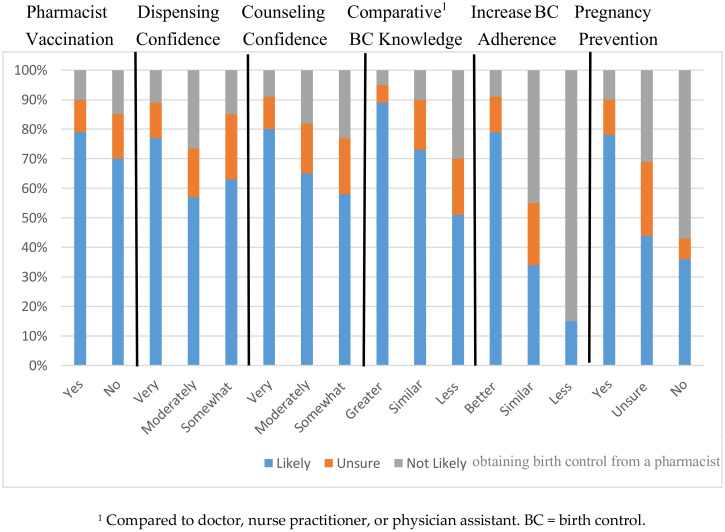
Likelihood of Obtaining Birth Control from a Community Pharmacist Based on Pharmacy Service Use and Opinions.

Previous use and quality assessment of pharmacy services and their impact on opinions about obtaining birth control from a pharmacy are depicted in Figure 2. Having received a vaccination from a pharmacist (*p* = 0.01), having greater confidence in pharmacist prescription dispensing (*p* = 0.01) and counseling (*p* = 0.000), and believing pharmacists have more knowledge than their doctor, nurse practitioner, or physician assistant (*p* = 0.000) were associated with greater likelihood of getting their birth control from a pharmacist.

Most female college students thought that receiving birth control from their pharmacist would definitely (59.5%) and probably (30.5%) increase their birth control adherence, and would definitely (63.0%) and probably (27.0%) decrease unintended pregnancies. The greater the belief of an impact of pharmacists prescribing birth control on increasing birth control adherence (*p* = 0.000) and decreasing unintended pregnancies (*p* = 0.000), the greater the likelihood of receiving their birth control from a community pharmacist.

#### 3.5.2. Pharmacist Attributes and Likelihood of Using a Pharmacist to Prescribe Birth Control

Students ranked personal attributes of a pharmacist in the 8.4 to 8.9 (out of 10) range with some aspects of the pharmacy and getting birth control in that environment lower (7.4 to 8.7). The results are shown in Table 4.

Using the nine attributes in Table 4, an ordered logistic regression analysis was done with the variable female college students’ likelihood of getting their birth control filled at a community pharmacy. The scale for likelihood of getting their birth control filled at a community pharmacy was reversed to aid in the interpretation of the odds ratios. The model was significant in describing pharmacist prescribed birth control (*p* < 0.0001). The attributes trustworthiness (OR 1.267, CI 1.130–1.420, *p* < 0.001), approachability (OR 1.127, CI 1.005–1.265, *p* = 0.041) and visit expense (OR 0.890, CI 0.824–0.960, *p* = 0.003) were statistically significant in predicting likelihood of using a pharmacist as a primary provider for contraceptives. The greater the perception of trustworthiness and approachability the students had for the pharmacist, the greater their likelihood to use the pharmacist as a primary provider for birth control. The lower the students perceived the pharmacy visit expense to be, the more likely they would use the pharmacist as a primary provider of birth control.

## 4. Discussion

This study provided evidence that women are interested in pharmacists prescribing birth control. Seventy-three percent of Michigan female college students felt they were likely to obtain their birth control from a community pharmacist. They felt the community pharmacist visit for birth control would be more convenient, save time, and make obtaining birth control easier. They also felt this pharmacy service would help them continue and be more adherent to their birth control, and prevent unintended pregnancies. The predominant concerns the students had were not getting regular Pap smears and screenings and potentially being prescribed the wrong birth control. These advantages and concerns are similar to other studies [14,15,16,17,18,19,20,21,22,23,24]. Many women do not know that a Pap smear and breast exam are not required for birth control use [2].

In terms of targeting this service to select groups of college students, we found across all our student characteristics to have high support for obtaining birth control in a community pharmacy, making the service generalizable across diverse students. Overall, at least 70% of the female college students across the various student characteristics stated they would use pharmacist birth control services, a measurement of acceptability. No statistically significant differences were seen between race and ethnic groups, religion/spirituality groups, residence while going to college, size of city living in during childhood, political party, type of college, student enrollment status, employment, and medical or medication insurance coverage. Students from a foreign country (i.e., student visa or immigrant status) were more likely to use this service than American born and naturalized citizen students, which might be related to lack of a primary care provider, health insurance, and or transportation, or non-prescription access to contraception in their birth country. Healthcare students were more likely than non-healthcare students to use a pharmacist for birth control, which might reflect greater understanding of pharmacist education and training and subsequent medication expertise. Variability within the healthcare students existed with pharmacy students having the highest, medical students in the middle, and nursing students the lowest likelihood. These differences could be related to territoriality and profession-centrism regarding writing prescriptions for patient management [25]. No differences existed within the various non-healthcare student disciplines, which could mean similar awareness of pharmacist skills and training. Graduate/professional degree students were more likely to use their pharmacist for birth control than undergraduate and associate degree students, which could represent healthcare professional awareness, maturity, and independence in health decisions. We thought more risky sexuality practices might increase willingness to access birth control from a pharmacist, but variability in willingness to use this service did not vary by type of relationship, number of unprotected intercourse events per year, condom use, or worry about getting pregnant.

Our study is the first study with a diverse female college participant cohort to assess opinions about pharmacists prescribing birth control, a group which could use this service. We chose this cohort since college students are in the age groups with the highest unintended pregnancy rates [1]. Another college student study surveyed California student pharmacists and almost all the student pharmacists (96%) wanted to provide this service [23]. They were not asked if they would use this service. These student pharmacists identified similar advantages as our college students about this pharmacy service. They had more concerns than our students such as the adequacy of pharmacist training, lack of a medical chart with concerns about patient data adequacy, and no incentive for pharmacists providing this service. By knowing more about pharmacy operations, student pharmacists could conceptualize this service differently than non-student pharmacists. Teenagers and young adults also liked the privacy and convenience of the community pharmacist prescribed birth control [16,19,20,21]. They also had some similar concerns to the college students such as pharmacists’ abilities, minimal patient specific data available, and limited birth control products. Similar to our student cohort, analysis of public commentary to news articles from women and men in Oregon supported pharmacists prescribing birth control, citing the service would be an asset and allow women to make reproductive health decisions [26]. Within the comments were similar concerns about incorporating this service into pharmacy operations, importance of physician patient relationship, safety issues, and insurance coverage.

Unintended pregnancy rates are higher in minority women and those living in poverty [2]. In our study, many minority women (68–83%) would get birth control from a pharmacist. Also, in our study, 69% of the female college students with a public insurance plan would get their birth control at a pharmacy. How many of these students would use this service is unknown. In Oregon, a state in which Medicaid offers and pays for this service, 10% of women with this insurance benefit chose a pharmacy to obtain their new birth control prescriptions [26]. In California, Oregon, and New Mexico, pharmacists offered birth control prescribing in rural and urban community pharmacies [27,28], which would align with our female college students in rural, small, and urban residences willing to get birth control from a community pharmacist.

Past experiences with pharmacists influenced the female college students’ likelihood of having a pharmacist prescribe birth control to them in a community pharmacy. Using pharmacist birth control services in the future varied based on past pharmacy services used and confidence in pharmacists. Having received a vaccination from a pharmacist and being more confident with pharmacists filling prescriptions and providing counseling were associated with a greater likelihood of obtaining birth control from a community pharmacist. About one third of the students (37.3%) thought pharmacists knew more about birth control than primary care providers, which could be influenced by the high percentage of pharmacy students in the database. Students’ rankings of pharmacist attributes were very positive, at least 8.4 or higher on a 10-point scale. Pharmacy attributes such as privacy, time, and cost were lower but still above 7.4 on a 10-point scale. Pharmacist trustworthiness, approachability, and visit expenses significantly influenced their likelihood of using birth control pharmacists. In the opinion-based studies about pharmacist prescribing, women of all ages would use or support this service, but they do express concerns as well [14,15,16,17,18,19,20,21,22,23,24,25,26,27,28]. Our research adds that prior use of pharmacy services and perceptions of pharmacist attributes influence these opinions.

Our study had a few limitations. The results represent one Midwest state and only college student opinions, which might not represent other states or women of different ages. Within all demographics, likelihood to use pharmacist birth control prescribing was high. The chance to win one of two $250 gift cards was most likely not an incentive for survey completion. Since the survey incentive was insignificant, respondent bias could exist with those more positive or opposed more likely to complete the survey. Recruitment was challenging with only 857 completed surveys after mass emailing, social media posts, and face to face recruiting at two major universities and two community colleges. Difficulty recruiting for another public perception of pharmacists prescribing birth control also was noted in a study using social media to recruit women of any age [19]. Only 2.2% of 44,994 social media advertisements on Facebook were clicked on by viewers, and only 28.9% of those clicks resulted in opening the survey. Although the survey had good diversity with most demographic variables, race and ethnicity diversity were limited with 83% of the students being white and 4.4% being Hispanic. In Michigan, 66% of the undergraduate college students are white, 12.1% black, 5.3% Hispanic, 4.6% Asian, 0.6% American Indian or Alaska Native, 3.5% of two or more races [29]. We attempted targeting minority female college organizations and courses, but these strategies were not successful. Our study represented the group with highest number of unintended pregnancies (60% were 20–24 years old) but other groups such as teenagers (19%) and those 30 years and older (8%) were less represented. The survey was long because we had 4 areas to explore. Survey burden might explain the number of students that opened the survey without answering anything besides their gender or did not finish or skipped certain sections. Our pre-study student sample stated the survey was doable.

Some but not all students might have been aware of pharmacists prescribing birth control prior to the study so different degrees of previous opinion development. Many student pharmacists could have learned about this service in class or at national and state pharmacy meetings. Learning about this service might have occurred in other curriculums. Medical students might have discussed the American College of Obstetrics and Gynecology preference for over-the-counter birth control, which could have influenced their opinions on pharmacists prescribing contraception. Little is known about the coverage of this new pharmacy service in other healthcare and non-healthcare (e.g., law, women’s studies) student curriculums. At the initiation of this survey, this pharmacy service was not available in Michigan. Now one large pharmacy chain in Michigan is offering this service through a collaborative practice agreement. The pharmacy chain started its service the month after our recruitment ended, which was the month Covid-19 hit Michigan. Prior to Covid-19, minimal marketing had occurred and during Covid-19, pharmacists prescribing birth control was not the focus of pharmacy patient health care, so most likely this new pharmacy provider of birth control had little impact on students’ opinions.

## 5. Conclusions

Many female college students in Michigan are receptive to pharmacists prescribing birth control in a community pharmacy. They feel this service will make getting and adhering to birth control easier and decrease their worries about getting pregnant. Their positive attitude results combined with reported prevention of unintended pregnancies and decreased health care costs outcomes in Oregon [17] support all states offering pharmacists prescribed birth control services. Further research is needed to confirm women’s and societal benefits and use of pharmacists prescribing birth control.

## Figures and Tables

**Table 1:** Female College Student Characteristics and Likelihood of Obtaining Birth Control from a Community Pharmacist.

Characteristic	No. Stds.	Percent of Students	Percent Likely to Obtain BC from a Pharmacist	*p*-Value	Characteristic	No. Stds.	Percent of Students	Percent Likely to Obtain BC from a Pharmacist	*p*-Value
Race	834			0.962	Healthcare student	528	63.70%	77.5% ^1^	0.044
White	692	83.0%	75.4%		Pharmacy	274	51.90%	83.3%	
Asian	67	8.3%	69.7%		Nursing	72	13.60%	66.2%	
Black/African American	40	4.8%	68.4%		Physician	33	6.30%	74.2%	
More than one race	22	2.6%	71.4%		Physician Assistant	17	3.20%	76.5%	
American Indian or Alaskan	7	0.8%	83.3%		Social worker	16	3.00%	71.4%	
Native					Other	116	25.00%	72.3%	
Other	5	0.7%	80.0%		Non-healthcare student	298	36.30%	69.3% ^1^	0.599
Ethnicity	833			0.741	Business	71	23.80%	61.8%	
Arab American	56	6.7%	74.5%		Law	33	11.10%	75.8%	
Hispanic/Latina	37	4.4%	82.4%		Engineering	32	10.70%	67.7%	
Religion/Spirituality	859			0.547	Education	32	10.70%	65.5%	
Christianity	486	58.9%	75.8%		Science	26	8.70%	76.0%	
Agnostic	122	14.8%	74.2%		Arts, music, theater	23	7.70%	69.6%	
Atheist	117	14.2%	73.7%		Liberal arts	22	7.40%	68.4%	
Islam	52	6.3%	72.5%		Humanities	13	4.40%	92.3%	
Hinduism	12	1.5%	66.7%		Communications	10	3.40%	80.0%	
Buddhism	7	0.8%	66.7%		Computers and	10	3.40%	66.7%	
Judaism	6	0.7%	66.7%		information				
Other	18	2.8%	77.3%		technology				
Citizenship	833			0.001	Other	28	9.30%	63.0%	
American born	765	91.8%	74.5%		Institution type	763			0.085
Naturalized citizen	43	5.2%	76.2%		University and state	713	92.20%	76.40%	
Immigrant	10	1.2%	77.8%		Community college	50	7.80%	63.0%	
Student visa	10	1.2%	100%		Student enrollment	833			0.555
Other	5	0.6%	20.0%		Full time	750	90.00%	74.4%	
Residence during school	832			0.415	Part time	74	8.90%	77.5%	
Apartment	228	27.4%	77.1%		No degree, fun class	9	1.10%	75.0%	
With parents or family	213	25.6%	71.6%		Degree	833			0.017
House	204	24.5%	74.1%		Graduate	436	52.30%	79.4%	
Dormitory	108	13.0%	82.4%		Undergraduate	274	32.90%	67.4%	
University college	78	9.4%	65.8%		Associate	88	10.60%	73.2%	
apartment					Other	35	4.20%	75.0%	
Homeless	1	0.1%	100%		Employment	859			0.529
City size of childhood	859			0.405	Part time	544	65.30%	74.7%	
Detroit and suburb	205	24.6%	70.2%		Not working	192	23.00%	76.6%	
Urban cluster	178	21.3%	81.1%		Full time	97	11.60%	70.0%	
Rural town or village	170	20.4%	76.5%		Health insurance	832			0.199
Small city	112	13.4%	69.2%		Parents’ plan	545	65.50%	76.3%	
Medium city	71	8.5%	73.9%		Public plan	130	15.60%	68.5%	
Large city	64	7.7%	74.2%		Private plan	125	15.00%	74.2%	
Foreign born	20	2.4%	80.0%		None	21	2.50%	77.8%	
Remote area	14	1.7%	78.6%		Unknown	11	1.30%	60.0%	
Childhood residence	832			0.876	Medication insurance	831			0.313
Lower peninsula	688	82.4%	74.3%		Private plan	523	62.90%	77.0%	
Upper peninsula	76	9.1%	77.8%		Public plan	125	15.00%	70.3%	
Outside of Michigan	45	5.4%	72.1%		Unknown	125	15.00%	69.4%	
Foreign country	26	3.1%	80.8%		None	58	7.00%	72.7%	
					Political party	830			0.742
					Democrat	371	44.70%	73.4%	
					None	194	23.40%	74.3%	
					Republican	157	18.90%	79.3%	
					Independent	96	11.60%	70.7%	
					Other	4	1.40%	83.3%	

^1^*p*-value for the difference between health care and non-health care students = 0.036. BC = birth control.

**Table 2:** Advantages and Concerns About Pharmacists Prescribing Birth Control in a Community Pharmacy.

Advantages ^1^	Number of Students	Percent of Students	Concerns ^1^	Number Students	Percent of Students
More convenient than visiting doctor	773	90.0%	Not get regular Pap smears and screening	690	80.3%
Save time	695	80.9%	Prescribed wrong birth control	377	43.9%
Easier to get birth control	686	79.9%	Use birth control incorrectly	212	24.7%
Less likely to run out of birth control	617	71.8%	Encourage teens to have sex earlier	209	24.3%
Better hours than doctor	560	65.2%	Insufficient skills of pharmacists	113	13.2%
Cost less than going to doctor	538	62.6%	Insufficient knowledge of pharmacists	78	9.1%
More private	125	12.3%	Believe birth control should not be used	16	1.9%

^1^ Options listed in the survey.

**Table 3:** Sexual Behaviors and Likelihood of Obtaining Birth Control from a Community Pharmacist.

Characteristic	Number Students	Percent of Students	Likelihood of Obtaining Birth Control from a Pharmacist			
			Extremely or Moderately	Unsure	Not Very Likely or Not at All	*p*-Value
Sexually active with a man	570	68.8%	84.0%	7.5%	8.5%	0.027
Relationship status	829					0.906
Committed relationship not living together	318	38.4%	73.8%	13.9%	12.3%	
No relationship, not sexually active	191	23.0%	75.3%	11.0%	13.7%	
Committed relationship, living together	116	14.0%	75.7%	12.2%	12.2%	
No relationship, sexually active	106	12.8%	73.8%	16.5%	9.7%	
Married	87	10.5%	75.0%	11.3%	13.8%	
Relationship with a woman	6	0.7%	83.3%	16.7%	0.0%	
Other	2	0.6%	50.0%	50.0%	0.0%	
Times unprotected intercourse past year if sexually active	814					0.044
Never	306	32.4%	75.8%	10.1%	14.1%	
One time	47	6.5%	88.9%	6.7%	4.4%	
A few times	143	19.8%	69.3%	15.7%	15.0%	
Most of the time	94	13.0%	73.6%	16.5%	9.9%	
Every time	132	18.3%	80.3%	13.4%	6.3%	
Condom use ^1^	815					
Never	240	36.9%	77.8%	11.5%	10.7%	0.761
<50% of the time	129	19.8%	75.2%	13.6%	11.2%	
50–75%	31	4.8%	75.9%	13.8%	10.3%	
76–90%	47	7.2%	72.7%	20.5%	6.8%	
90–99%	87	13.4%	77.9%	14.0%	8.1%	
100%	117	18.0%	75.2%	9.7%	15.0%	
Worried about getting pregnant	819					0.638
Very worried	40	4.9%	81.6%	10.50%	7.9%	
Moderately worried	92	11.2%	79.1%	9.90%	11.0%	
Somewhat worried	184	22.5%	77.1%	11.20%	11.7%	
Not worried	328	40.0%	72.9%	15.50%	11.7%	
Not in a sexual relationship	175	21.4%	71.9%	12.60%	15.6%	
Emergency contraception use	281	34.6%	68.8%	18.80%	12.5%	Not done

^1^ Of sexually active students.

**Table 4:** Student Ratings on Pharmacist Attributes in Birth Control Prescribing Scenario.

Pharmacist Attribute	Number of Students	Mean	Standard Deviation
Trustworthiness	848	8.5	1.7
Approachability	848	8.4	1.9
Respectfulness	846	8.7	1.7
Knowledge of birth control	848	8.9	1.6
Ability to provide education on birth control	848	8.4	1.9
Ability to provide privacy	840	7.8	2.3
Access (ease of making an appointment)	843	8.7	1.9
Time (visit, prescription fill, and travel)	849	7.9	2.6
Expense (all costs related to visit and prescription)	850	7.4	2.8

## Appendix A

### Survey

**Pharmacists Prescribing Birth Control in a Community Pharmacy**

Q1 I wish to continue with the survey

○ I do not wish to continue with this survey.

*Skip To: End of Survey If Research Information Sheet Pharmacists Prescribing Bi … = I do not wish to continue with this survey.*

Q2 What is your birth sex?

○ Female
○ Male
○ Intersex (born with both female and male genitalia)

*Skip To: Q3 If What is your birth sex? = Male*

*Skip To: End of Block If What is your birth sex? = Female*

*Skip To: End of Block If What is your birth sex? = Intersex (born with both female and male genitalia)*

Q3 Your opinions on this pharmacy service are important, however for this survey only female students are eligible to participate.

*Skip To: End of Survey If Your opinions on this pharmacy service are important, however for this survey only female student … () Is Displayed*

**Health Care Experiences**

Q4 Recall a previous experience with, or imagine visiting, a gynecologist or clinic provider for a prescription for birth control. How would you rate each of the below topics related to that visit on a scale of 1 being the lowest to 10 being the highest?

	1	2	3	4	5	6	7	8	9	10
Provider trustworthiness	○	○	○	○	○	○	○	○	○	○
Provider approachability	○	○	○	○	○	○	○	○	○	○
Provider respecting me	○	○	○	○	○	○	○	○	○	○
Provider knowledge of birth control	○	○	○	○	○	○	○	○	○	○
Birth control education received	○	○	○	○	○	○	○	○	○	○
Privacy	○	○	○	○	○	○	○	○	○	○
Ease of making an appointment	○	○	○	○	○	○	○	○	○	○
Time commitment—all time related to getting the birth control prescription, e.g., calling to get an appointment, waiting time to see provider, provider interaction, getting the prescription filled	○	○	○	○	○	○	○	○	○	○
Expense—all costs related to getting the birth control prescription, e.g., clinic visit, prescription cost, travel	○	○	○	○	○	○	○	○	○	○

Q5 Imagine you want to begin taking birth control while away at college. You opt to go see a pharmacist at your community pharmacy, who now has the legal right to prescribe birth control. The pharmacist will ask you a series of questions and then determine which birth control would be best for you. The pharmacist would write your birth control prescription that could be filled that day in the pharmacy. How would you rate each of the below topics related to that visit with the pharmacist on a scale of 1 being the lowest to 10 being the highest?

	1	2	3	4	5	6	7	8	9	10
Pharmacist trustworthiness	○	○	○	○	○	○	○	○	○	○
Pharmacist approachability	○	○	○	○	○	○	○	○	○	○
Pharmacist respecting me	○	○	○	○	○	○	○	○	○	○
Pharmacist knowledge of birth control	○	○	○	○	○	○	○	○	○	○
Birth control education received	○	○	○	○	○	○	○	○	○	○
Privacy	○	○	○	○	○	○	○	○	○	○
Ease of making an appointment	○	○	○	○	○	○	○	○	○	○
Time commitment—all time related to getting the birth control prescription, e.g., calling to get an appointment, waiting time to see provider, provider interaction, getting the prescription filled	○	○	○	○	○	○	○	○	○	○
Expense—all costs related to getting the birth control prescription, e.g., clinic visit, prescription cost, travel	○	○	○	○	○	○	○	○	○	○

**Pharmacy Services and Evaluation**

Q6 Have you ever received a vaccination from a pharmacist in a community pharmacy?

○ Yes
○ No

Q7 How confident are you in your community pharmacist accurately filling your prescription medications?

○ Extremely confident
○ Very confident
○ Moderately confident
○ Somewhat confident
○ Not confident

Q8 How confident are you in your community pharmacist counseling you on your prescription medications?

○ Extremely confident
○ Very confident
○ Moderately confident
○ Somewhat confident
○ Not confident

**Pharmacists Prescribing Birth Control Opinions, Advantages, and Barriers**

Q9 Please rate the following statement on your level of agreement. Pharmacists know more about birth control than my doctor, nurse practitioner, or physician assistant?

○ Strongly agree
○ Agree
○ Neither agree nor disagree
○ Disagree
○ Strongly disagree

Q10 If you could get your birth control pills at a community pharmacy without first visiting your doctor, nurse practitioner, or physician assistant, how likely would you be to do so?

○ Extremely likely
○ Moderately likely
○ Unsure
○ Not very likely
○ Not likely at all
○ Never, I do not use birth control

Q11 Do you feel it would be easier for a woman to stay on birth control if her pharmacist could prescribe her birth control prescriptions?

○ Definitely yes
○ Probably yes
○ Maybe
○ Probably not
○ Definitely not

Q12 Do you feel it would be easier for a woman to prevent unwanted pregnancies if her pharmacist could prescribe her birth control prescriptions?

○ Definitely yes
○ Probably yes
○ Maybe
○ Probably not
○ Definitely not

Q13 What would be the advantages of pharmacists prescribing birth control compared to getting these medications from your doctor or clinic? (Select all that apply)

○ It would be more convenient than visiting my doctor
○ I would be less likely to run out of birth control
○ It would be more private
○ It would cost me less than going to the doctor
○ The pharmacy has better hours than my doctor
○ It would be easier to get birth control
○ It would save me time
○ Other advantages, please describe_____________________________________
○ None

Q14 What concerns do you have about pharmacists writing prescriptions for birth control? (select all that apply)

○ Women might not regularly get Pap smears and screening.
○ Women might be prescribed the wrong birth control.
○ Women might use birth control incorrectly.
○ It could encourage teens to have sex earlier if birth control is easier to get.
○ Pharmacists do not have enough knowledge to prescribe birth control.
○ Pharmacists do not have the skills to assess safety of birth control for women.
○ I don’t believe that birth control pills should be used by anyone.
○ Other concerns, please describe ______________________________________
○ None

**Personal Characteristics**

Q15 What is your age (years)? _____________________________________

Q16 What is your race?

○ American Indian or Alaskan Native
○ Asian
○ Black/African American
○ Hawaiian or Pacific Islander
○ White
○ More than one race
○ Other, please describe ________________________________________________

Q17 What is your ethnicity?

○ Arab
○ Hispanic/Latina
○ Neither

Q18 What is your spirituality or religion that you currently practice?

○ Agnostic
○ Atheist
○ Buddhism
○ Christianity
○ Hinduism
○ Islam
○ Judaism
○ Monotheism
○ Sikhism
○ Other, please list ________________________________________________

Q19 Do your religious beliefs influence your use of birth control for the purpose of preventing an unwanted pregnancy? This question does not refer to other uses of birth control such as acne, endometriosis, etc.

○ I have no religious beliefs therefore no impact on my decisions.
○ Because my religion opposes birth control, I would not use it.
○ Per my religion or culture, I would only use birth control after I am married.
○ Per my religion or culture, I would only use birth control if ok with my partner.
○ I would use birth control even though my religion opposes it.
○ My religion is not opposed to birth control and therefore I could use birth control.

Q20 What is your citizenship?

○ American born
○ Immigrant
○ Naturalized citizen
○ Student visa
○ Other status, please describe _____________________________________________

Q21 Where do you live during school?

○ Dormitory
○ University/college apartment
○ Apartment
○ House
○ With parents or family
○ Homeless

Q22 Describe the size of the city where you grew up?

https://www.michigan-demographics.com/cities_by_population

○ Remote area, e.g., unincorporated city
○ Rural town or village, <2500 people, e.g., Cass City; St. Ignace, Harrison
○ Urban cluster (2500–24,999 people) e.g., Escanaba, Big Rapids, Traverse City, Ishpeming
○ Small city (25,000–49,999 people) e.g., Midland, Bay City, Port Huron, Muskegon
○ Medium city (50,000–99,999 people) e.g., Flint, Kalamazoo, Battle Creek
○ Large city (100,000–249,000 people) e.g., Lansing, Grand Rapids, Ann Arbor
○ Detroit and Suburbs (250,000 or more people) e.g., Detroit, Livonia, Rochester Hills
○ In a foreign country

Q23 Which part of Michigan did you grow up?

○ Upper peninsula
○ Lower peninsula
○ Outside of Michigan in the US
○ Outside of Michigan in a foreign country

Q24 What type of student are you?

○ Part time
○ Full time
○ Just taking classes for no degree

Q25 What type of degree are you seeking?

○ High school student taking college classes
○ Certificate
○ Associate degree
○ Undergraduate degree
○ Graduate degree
○ Taking prerequisites for a future degree program
○ Just taking classes for fun

*Skip To: Q29 If What type of degree are you seeking? = High school student taking college classes*

*Skip To: Q29 If What type of degree are you seeking? = Just taking classes for fun*

Q26 Are you in a health professional program or taking classes to get into a health professional program?

○ Yes
○ No

*Skip To: Q28 If Are you in a health professional program or taking classes to get into a health professional prog … = No*

Q27 Which health professional curriculum are you studying?

○ Allied health, please state which one _______________________________________
○ Dentistry
○ Nursing
○ Occupational Therapy
○ Optometry
○ Pharmacy
○ Physical Therapy
○ Physician
○ Physician Assistant
○ Social Worker
○ Other health profession, please describe ________________________________________________

*Skip To: Q29 If Which health professional curriculum are you studying? = Allied health, please state which one*

*Skip To: Q29 If Which health professional curriculum are you studying? = Dentistry*

*Skip To: Q29 If Which health professional curriculum are you studying? = Nursing*

*Skip To: Q29 If Which health professional curriculum are you studying? = Occupational Therapy*

*Skip To: Q29 If Which health professional curriculum are you studying? = Optometry*

*Skip To: Q29 If Which health professional curriculum are you studying? = Pharmacy*

*Skip To: Q29 If Which health professional curriculum are you studying? = Physical Therapy*

*Skip To: Q29 If Which health professional curriculum are you studying? = Physician*

*Skip To: Q29 If Which health professional curriculum are you studying? = Physician Assistant*

*Skip To: Q29 If Which health professional curriculum are you studying? = Social Worker*

*Skip To: Q29 If Which health professional curriculum are you studying? = Other health profession, please describe*

Q28 What area are you studying?

○ Arts, Music, Theater
○ Business
○ Communications
○ Computers and or Information Technology
○ Education
○ Engineering
○ Humanities
○ Law
○ Liberal Arts
○ Prerequisites for a health professional degree
○ Science
○ Other area of study, please describe ________________________________________________

Q29 What is your current employment history?

○ Not working
○ Part time
○ Full time

Q30 Which political party do you identify the most with?

○ Democrat
○ Independent
○ Republican
○ Other affiliation, please state _____________________________________________
○ None

Q31 What type of health insurance do you have?

○ None
○ Under parents’ plan
○ Private
○ Public, e.g., Medicaid, Medicare
○ I don’t know

Q32 Do you have medication or health insurance that would cover the cost of birth control?

○ No
○ Yes, public assistance
○ Yes, private plan
○ I don’t know

**Sexual and Reproductive History**

Q33 Describe your current relationship status with a man or partner(s)?

○ Married
○ Committed relationship: not living together
○ Committed relationship: living together
○ Relationship with multiple male partners
○ Relationship with a woman
○ Other relationship, please describe_________________________________________
○ Not in a relationship and not sexually active
○ Not in relationship but sexually active

Q34 Are you currently sexually active with a male partner?

○ Yes
○ No

*Skip To: Q36 If Are you currently sexually active with a male partner? = Yes*

Q35 Have you been sexually active with a male partner in the past?

○ Yes
○ No

Q36 How old were you when you had intercourse for the first time?

○ Age (years) ________________________________________________
○ Have not had intercourse

Q37 How many male sexual partners have you had in your life? _____________________

Q38 On average, how many times do you have intercourse per week?

○ Times per week ________________________________________________
○ Do not have intercourse
○ Not in a relationship now

Q39 How often have you had unprotected intercourse in the past 12 months?

○ Never
○ One time
○ A few times
○ Most of the time
○ Every time
○ No intercourse in the last year

Q40 Are you worried about getting pregnant?

○ Very worried
○ Moderately worried
○ Somewhat worried
○ Not worried
○ Not in a sexual relationship

Q41 How often do you use condoms when having intercourse?

○ Never
○ <50% of the time
○ 50–75% of the time
○ 76–90% of the time
○ 90–99% of the time
○ 100% of the time
○ Not sexually active

Q42 What do you currently use for birth control? (Select all that apply)

○ None
○ Not needed, I am not sexually active.
○ Not needed, I am pregnant.
○ Not needed, I am incapable of becoming pregnant due to medical diagnosis or physical condition (e.g., hysterectomy, abnormal organs, sterility).
○ Not needed, I am postmenopausal.
○ Oral pills
○ Shots
○ Implant
○ Ring
○ IUD
○ Condom
○ Spermicide
○ Condom and spermicide
○ Diaphragm
○ Withdrawal
○ Rhythm method
○ Abstinence
○ Emergency contraception

Q43 If you are using hormonal birth control (eg, pills, implants, rings, etc.), why are you using this medication? (Select all that apply)

○ Birth control
○ Acne
○ Menstrual problems such as endometriosisi, PMS, PMDD, painful periods, excessive monthly bleeds
○ Other use, please describe
○ Not using hormonal birth control

Q44 How many times have you used emergency contraception (Plan B, Preven, etc.)?

○ Never used
○ Number of times ________________________________________________

Q45 How many times have you been pregnant?

○ Never been pregnant
○ Number of pregnancies ________________________________________________

Q46 Where do you currently go for routine gynecological care such as Pap smear?

○ Campus health
○ Planned parenthood
○ Private physician, nurse practitioner, physician assistant
○ Urgent care
○ Emergency room
○ I do not routinely get Pap smears

Q47 Where do you currently go for a vaginal infection? (Select all that apply)

○ Campus health
○ Planned parenthood
○ Private physician, nurse practitioner, physician assistant
○ Urgent care
○ Emergency room
○ Pharmacy
○ I have never had a vaginal infection

Q48 What University or College do you attend?

○ Bay De Noc Community College
○ Ferris State University
○ Oakland Community College
○ Wayne State University
○ Other ____________________________

Q49 What city do you currently live in? _____________________

**Raffle Information and Thank You**

Q50 Thank you for participating in this study. We appreciate your time and responses. Your responses will be very helpful to understand the needs of women in Michigan and your opinions about a potential new women’s health service—pharmacists prescribing birth control in a community pharmacy.

Would you like to enter the raffle for a chance of receiving one of the two $250 gift cards? Your name and email will not be connected with your survey responses.

○ Yes enter me in the draw
○ Do not enter me in the draw
